# Spontaneous Proliferation of H2M-/- CD4 T Cells Results in Unusual Acute Hepatocellular Necrosis

**DOI:** 10.1371/journal.pone.0110516

**Published:** 2014-10-14

**Authors:** Jeong-su Do, William M. Baldwin, Booki Min

**Affiliations:** Department of Immunology, Lerner Research Institute, Cleveland Clinic Foundation, Cleveland, Ohio, United States of America; Boston University School of Medicine, United States of America

## Abstract

Naïve CD4 T cells are triggered to undergo spontaneous proliferation, a proliferative response induced in response to homeostatic stimulation, when exposed to severe lymphopenic environments. They spontaneously acquire proinflammatory effector phenotypes, playing a major role in inducing chronic inflammation in the intestine that is believed to be induced by T cell recognition of commensal antigens. While the antigens inducing the T cell responses and inflammation are being extensively investigated, the role of clonality of T cells involved in this process remains poorly understood. In this study, we utilized naïve CD4 T cells isolated from B6 H2M−/− mice, in which MHCII molecules are complexed with a single CLIP molecule, and examined spontaneous proliferation and intestinal inflammation of CD4 T cells expressing limited T cell receptor repertoire diversity. We found that H2M−/− CD4 T cells undergo robust spontaneous proliferation, differentiate into IFNγ-producing Th1 type effector cells, and, most unexpectedly, induce severe acute hepatocellular necrosis. T cell interaction with MHCII molecule on cells of hematopoietic origin was essential to induce the pathology. Interestingly, B cells are fully capable of preventing necrotic inflammation via IL-10-independent and B7-H1-dependent mechanism. This could be a useful animal model to examine T cell-mediated liver inflammation and B cell-mediated immune regulation.

## Introduction

Maintaining lymphocyte homeostasis is a central process pivotal for both immunity and tolerance [1]. Dysregulation of the homeostatic process is thought to directly link to uncontrolled immune activation such as autoimmunity. Experimental T cell induced intestinal inflammation is a condition that T cell proliferation is triggered by homeostatic disturbance in response to otherwise harmless commensal (and self) antigens [2]. Proliferating cells differentiate into effector cells producing proinflammatory cytokines, mediating chronic inflammation in the target tissues, i.e., intestine [3], [4]. Polyclonal naïve CD4 T cells are typically used in this model, as good proportion of these cells is reactive (and possibly cross-reactive) to these antigens. While this is a useful animal model to study pathogenesis of T cell-induced colitis that resembles human inflammatory bowel disease (IBD), the exact contribution of T cell clonality during colitogenic T cell immune responses remains largely unknown.

H2M is a MHCII-like molecule that displaces the invariant chain-derived CLIP peptide bound onto MHCII molecules with peptides generated within the endosomes via exogenous pathways, displaying various exogenous peptide antigen:MHCII complexes available for T cells to respond [5]. MHCII molecules in mice deficient in H2M are still bound to the CLIP. As a result, CD4 T cells from these animals develop under the influence of a single peptide CLIP:MHCII complexes, generating mature CD4 T cells expressing limited TCR repertoire diversity [6]. Interestingly, those cells were found to proliferate in response to syngeneic APCs [6]–[8]. It was proposed that mature CD4 T cells selected by the single peptide ligand are highly reactive to self-peptides, but with low affinity [9]. Consistent with this notion, H2M−/− CD4 T cells undergo robust proliferation when transferred into sublethally irradiated B6 recipients [5]. On the other hand, they undergo slow cell division in H2M−/− hosts, which is completely absent in MHCII−/− condition [5]. However, their ability to undergo spontaneous proliferation and the subsequent development of intestinal inflammation has not formally been examined.

In this study, we examined spontaneous proliferation of naïve H2M−/− CD4 T cells in severe lymphopenic recipients. Consistent with the previous findings [5], [8], naïve H2M−/− CD4 T cells underwent robust spontaneous proliferation when transferred into Rag−/− recipients. Unexpectedly, however, the recipients rapidly developed an acute hepatocellular necrosis. T cells primarily became IFNγ-producing effector cells, and IFNγ was found crucial for the pathogenesis. More interestingly, the T cell-induced necrosis in the liver was completely abrogated by the presence of B cells, suggesting a regulatory role. B cell-mediated protection was independent of IL-10 produced by B cells. Instead, B cell expression of MHCII and B7-H1 appeared to be essential to mediate their protective role. Taken together, the current study proposes a new animal model to study T cell-mediated necrotic inflammation in the liver as well as B cell-mediated immune regulation.

## Results

### Naïve CD4 T cells with limited repertoire diversity undergo robust spontaneous proliferation and induce necrotic inflammation in the liver in syngeneic lymphopenic recipients

The lack of H2M impairs the displacement of invariant chain-derived CLIP peptide on MHCII molecules within the endosome [7], resulting in that surface MHCII molecules are primarily occupied by the CLIP peptide and that CD4 T cells developed in these animals are selected by the single ligand CLIP:MHCII complexes and express relatively limited repertoire diversity [10]. It was noted that those CD4 T cells express proliferative activity in the culture with syngeneic APCs [10], suggesting that these CD4 T cells may respond to syngeneic MHCII molecules complexed with diverse antigenic peptides.

**Figure 1:**
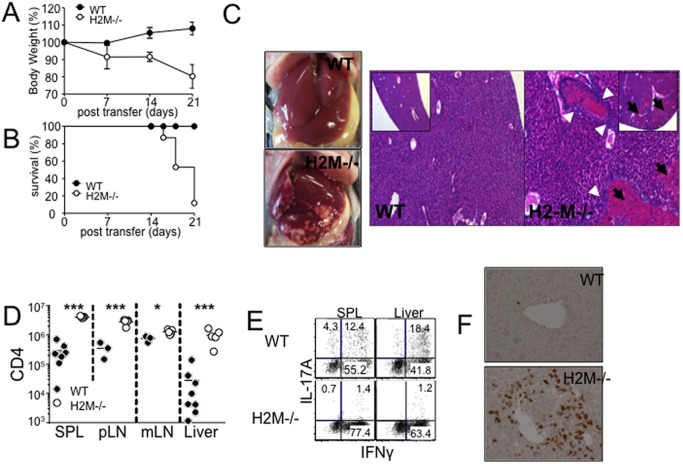
H2M−/− CD4 T cells induce severe necrotic inflammation in the liver. WT and H2M−/− naïve CD4 T cells were adoptively transferred into Rag−/− recipients. (A) Weight loss and (B) survival were monitored after CD4 T cell transfer. Data are the mean ± SD of 4–9 individually tested animals from 2–3 independent experiments. (C) Liver picture and H&E stain of liver, (D) absolute number of donor CD4 T cells in different tissue, and (E) intracellular cytokine expression of the donor cells from the indicated tissues were determined 7 days post transfer. (F) Immunohistochemistry analysis of the CD3+ cells in the liver. Each symbol represents individually tested animals from two independent experiments. *, p<0.05; ***, p<0.001.

When naïve CD4 T cells are transferred into severe lymphopenic hosts they undergo rapid spontaneous proliferation and develop into colitogenic effector cells producing IFNγ and/or IL-17 in response to antigens derived from commensal bacteria and self [2]. By transferring naïve phenotype (CD44^l^°^w^) Thy1.1+ H2M−/− CD4 T cells into Rag−/− recipients, the current study aimed at investigating the role of TCR repertoire diversity during spontaneous proliferation and the subsequent development of chronic intestinal inflammation. We first noticed that H2M−/− CD4 T cell recipients rapidly lost body weight, even starting 7 days post transfer (Fig. 1A), which is in good contrast with WT T cell recipients that typically develop chronic colitis 4–6 weeks after the transfer [11]. Moreover, almost all H2M−/− CD4 T cell recipients succumb to death by 3 weeks post transfer (Fig. 1B). While we found no signs of gut inflammation in these groups of animals, we instead observed that the liver of H2M−/− T cell recipients showed severe necrotic lesion by both gross and histologic examinations (Fig. 1C). Histology demonstrated numerous portal thrombi composed of central cores of platelets and fibrin surrounded by neutrophils and mononuclear leukocytes (Fig. 1C, white arrowhead). In addition, moderate periportal inflammatory infiltrates of mononuclear cells and some neutrophils were evident with minimal bile duct damage. These inflammatory lesions were associated with extensive areas of coagulative necrosis characterized by eosinophilic cytoplasm and many pyknotic and karyorrhectic nuclei (Fig. 1C, black arrow). Other tissues including the intestine, kidney, and lung appeared to be intact (data not shown). The liver of WT CD4 T cell recipients showed no signs of liver necrosis even during severe intestinal inflammation (Fig. 1C and data not shown).

To gain insights into the liver pathology induced by H2M−/− CD4 T cells, we examined CD4 T cells expansion and T cell infiltration into the liver. Overall expansion of H2M−/− CD4 T cells was dramatically higher in all tested lymphoid tissues. In particular, T cell accumulation in the liver was elevated >30-fold compared to that of WT CD4 T cells (Fig. 1D). While WT T cells typically differentiate into IFNγ- and IL-17- producing phenotype cells, H2M−/− CD4 T cells exclusively became IFNγ-producing cells (Fig. 1E). Th2 type cytokines including IL-4 and IL-10 were not found (data not shown). Immunohistochemistry also confirmed severe perivascular infiltration of the donor T cells in the liver (Fig. 1F).

### H2M−/− CD4 T cell spontaneous proliferation is not an oligoclonal response yet not all T cells are capable of inducing necrotic inflammation in the liver

**Figure 2:**
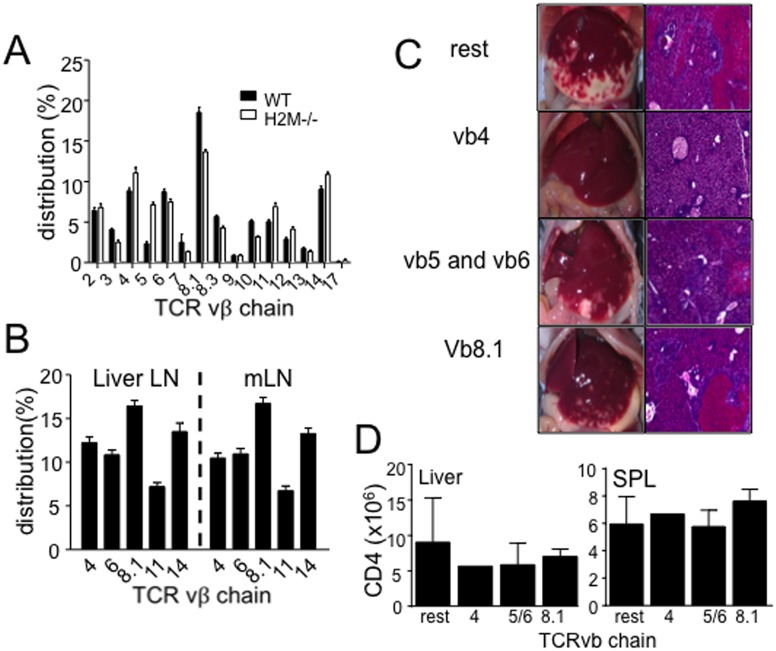
Distribution of TCR Vβ chain expression and liver inflammation induced by H2M−/− CD4 T cell subsets. (A) TCR Vβ chain was measured at 7 days after transfer. Cells were isolated from SPL and stained with anti-TCR Vβ antibodies. Data are the mean ± SD of 5 individually tested animals from two independent experiments. (B) Subtype of TCR Vβ chain was measured from liver draining LN and mLN. Data are the mean ± SD of 4 individually tested animals. (C) Induction of liver inflammation by Vβ+ subtype H2M−/− CD4 T cells. FACS purified Vβ4, Vβ5/6, and Vβ8.1 H2M−/− CD4 T cells were transferred into Rag−/− mice and analyzed liver pathogenesis at day 7. (D) Absolute number of donor CD4 T cells in the spleen and liver. Data are the mean ± SD of 2–3 individually tested animals.

Is this unusual response specific for liver specific antigen(s)? In case of autoimmune hepatitis, TCR repertoire analysis has demonstrated a significant skewing of the Vβ chain usage, an indication of oligoclonal expansion [12]. TCR Vβ chain distribution of T cells was thus compared after 7 days of transfer. Proliferating T cells were mostly polyclonal and indistinguishable between WT and H2M−/− T cells (Fig. 2A). Furthermore, we compared Vβ chain distribution of CD4 T cells isolated from the liver draining and gut draining lymph nodes. Again, no difference in the proportion of Vβ+ T cells was found between the tissues (Fig. 2B). To further validate this finding, we isolated naïve H2M−/− CD4 T cells based on the TCR Vβ expression. Naïve Vβ4+, Vβ5/6+, and Vβ8.1+ H2M−/− CD4 T cells were chosen based on the dominant distribution. The isolated T cells were then transferred into naïve Rag−/− recipients. T cells expressing other Vβ chain were also transferred (named ‘rest’). While relative T cell expansion was comparable regardless of the TCR Vβ chain (Fig. 2D), necrotic liver inflammation was dramatically different. For example, Vβ4+ T cell recipients did not develop any signs of inflammation (Fig. 2C). On the other hand, Vβ5/6+ and Vβ8.1+ T cell recipients developed moderate/severe inflammation in the liver (Fig. 2C). Mice that received T cells expressing the rest of TCR Vβ chain (rest) developed most severe form of necrotic inflammation (Fig. 2C). Therefore, there might be certain H2M−/− CD4 T cell clones involved in the development of inflammatory responses specifically in the liver, while other T cells undergo extensive spontaneous proliferation possibly in response to syngeneic APCs without obvious pathogenic effects in the liver [9].

### T cell interaction with wild type MHCII molecules is critical for the inflammation

**Figure 3:**
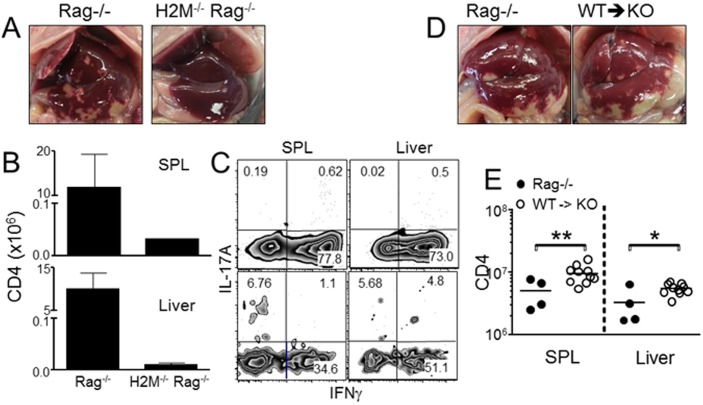
MHCII expression on recipients is required for liver inflammation mediated by H2M−/− CD4 T cells. (A) Liver pathogenesis in Rag−/− and H2M−/− Rag−/− recipients after H2M−/− T cell transfer. (B) Absolute numbers of donor CD4 T cells were analyzed 7 days post transfer. (C) IFNγ and IL-17A cytokine expression were determined by FACS analysis. (D and E) Lethally irradiated MHCII−/− Rag−/− mice were reconstituted with BM cells from Rag−/− (Rag−/− BM → MHCII−/− Rag−/−, WT → KO). FACS purified naive H2M−/− CD4 T cells were transferred into the reconstituted recipients after 6 weeks of BM reconstitution. Recipients were sacrificed 7 days post transfer. (D) Liver pathology and (E) absolute numbers of donor cells were assessed. All experiments were repeated twice and similar results were observed. Each symbol represents individually tested animals. *, p<0.05; **, p<0.01.

H2M−/− T CD4 T cells hyper-respond to syngeneic WT MHCII molecules expressed on APCs [10]. Supporting this notion, H2M−/− T cells transferred into H2M−/− Rag−/− recipients did not induce necrotic inflammation in the liver (Fig. 3A). Moreover, T cell expansion was substantially reduced in these recipients (Fig. 3B). Reduced expansion is not probably due to their inability to proliferate as they can proliferate efficiently in lymphopenic H2M−/− conditions [13]. Indeed, they efficiently differentiate into IFNγ producing effector cells in H2M−/− conditions (Fig. 3C). Therefore, H2M−/− T cell recognition of MHCII molecules complexed with highly diverse peptides is critical for both extensive proliferation and liver pathology.

MHCII expression by DCs is a key requirement for CD4 T cells to undergo spontaneous proliferation [14]. Whether T cell-induced inflammation requires MHCII expression by APCs, or alternatively, whether T cells recognize MHCII expressed by hepatocytes was next examined. Parenchymal cells do not usually express MHCII; however, under inflammatory conditions, hepatocytes often express MHCII and function as APCs to activate CD4 T cells [15]. To explore this possibility we generated bone marrow (BM) mixed chimeras. MHCII−/− Rag−/− recipient of Rag−/− BM cells similarly developed severe inflammation (Fig. 3D). T cell expansion was even more increased in this condition, compared to Rag−/− mice (Fig. 3E). Therefore, BM derived hematopoietic cells, most likely DCs, expressing MHCII molecules are sufficient to induce liver inflammation.

### B cells prevent H2M−/− T cell-induced inflammatory responses in the liver

**Figure 4:**
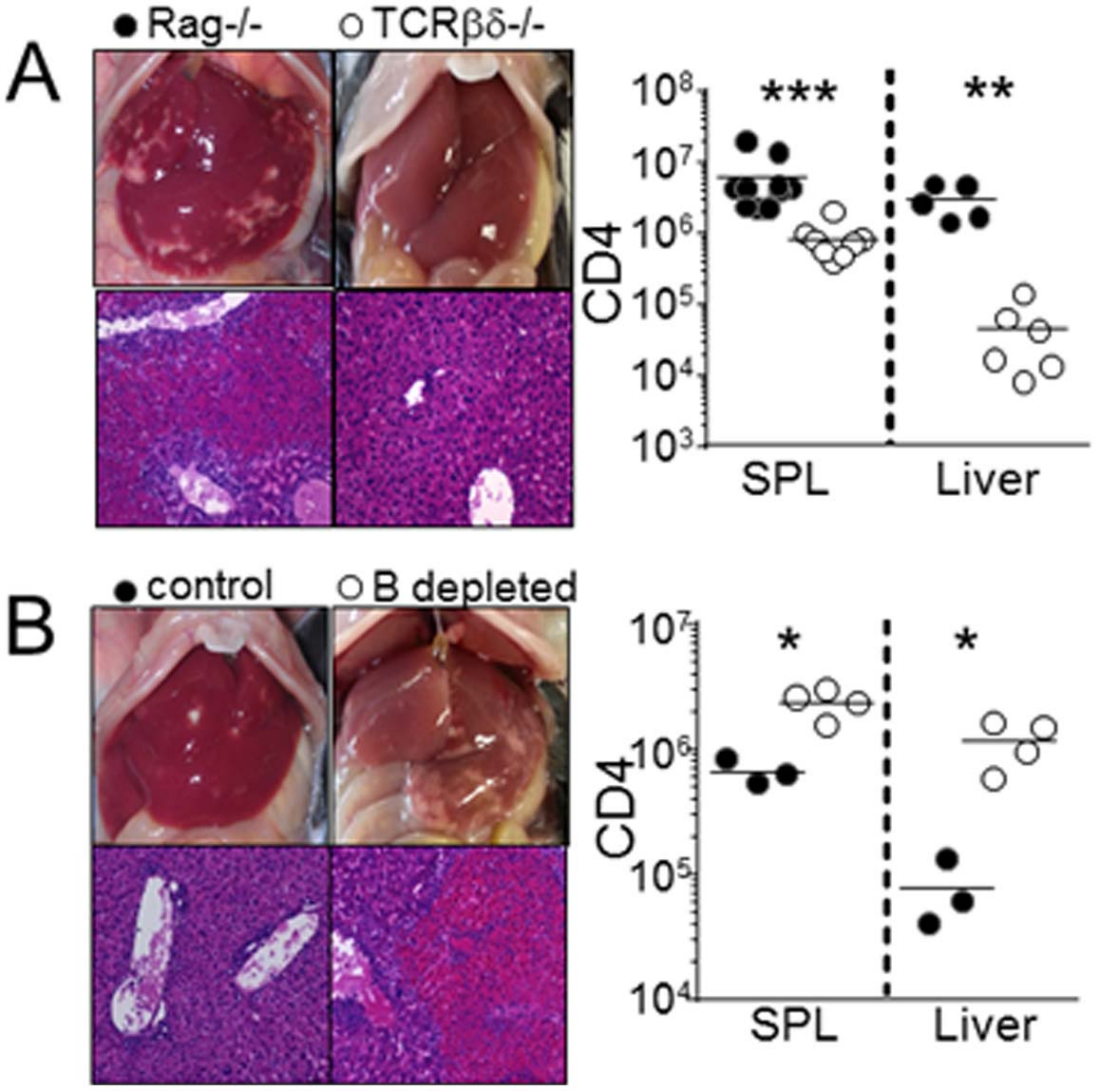
B cells prevent liver necrotic inflammation. (A) H2M−/− naïve CD4 T cells were transferred into Rag−/− and TCRβδ−/− recipients. Liver pathology and the absolute number of CD4 T cells in Rag−/− and TCRβδ−/− were examined at day 7. All experiments were repeated twice and similar results were observed. Each symbol represents individually tested animals. (B) Liver pathology and the absolute number of CD4 T cells in TCRβδ−/− and B cell depleted TCRβδ−/− recipients. Each symbol represents individually tested animals. *, p<0.05; **, p<0.01; ***, p<0.001.

We unexpectedly noticed that H2M−/− T cell-mediated necrotic inflammation in the liver was not found in other immunodeficient models, TCRβ−/− (data not shown) and TCRβδ−/− recipients (Fig. 4A). T cell expansion in general was significantly reduced in these ‘resistant’ recipients (Fig. 4A). Given that the major difference between Rag−/− and TCRβδ−/− conditions is the presence of B cells and that B cells can exert regulatory function by limiting autoimmune T cell responses [16], we hypothesized that B cells suppress necrotic inflammatory responses of H2M−/− CD4 T cells. To directly test the hypothesis, we depleted B cells in ‘resistant’ TCRβδ−/− recipients of H2M−/− CD4 T cells using anti-CD20 antibody [14]. Indeed, we found that B cell depletion in TCRβδ−/− mice completely restored necrotic inflammation in the liver (Fig. 4B). The overall T cell expansion was significantly elevated (Fig. 4B).

**Figure 5:**
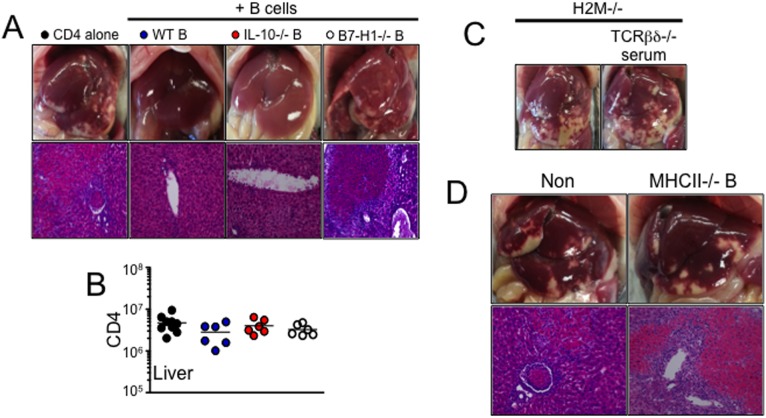
MHCII and B7-H1 on B cells are required to prevent liver inflammation. (A) H2M−/− naïve CD4 T cells were adoptively transferred into Rag−/− recipients together with 5×10^6^ WT, IL-10−/−, and B7-H1−/− B cells. Liver pathology and H&E stain (A) and the absolute numbers of CD4 T cells (B) in the liver were assessed 7 days after transfer. (C) H2M−/− naïve CD4 T cells were transferred into Rag−/− recipients. TCRβδ−/− serum was intravenously injected. (D) H2M−/− naïve CD4 T cells were adoptively transferred into Rag−/− recipients together with 5×10^6^ MHCII−/− B cells. All experiments were repeated twice and similar results were observed. Each symbol represents individually tested animals.

To further validate this finding, we adoptively transferred B cells into Rag−/− mice that received H2M−/− CD4 T cells. Supporting the hypothesis, B cell transfer fully prevented liver pathology in Rag−/− mice (Fig. 5A). Therefore, these results strongly suggest that B cells are capable of interfering with H2M−/− T cell-induced inflammation in the liver.

IL-10-producing B cells have regulatory functions in certain autoimmune disease models [16]. To directly test if IL-10 production by transferred B cells may prevent the inflammation, we transferred IL-10−/− B cells into Rag−/− recipients of H2M−/− T cells. As shown in Fig. 5A, IL-10−/− B cells were equally capable of preventing the inflammation, indicating that IL-10 production by B cells is dispensable. In murine experimental stroke model [17], it was shown that B7-H1 expressing B cells can inhibit activation of T cells and APCs in the central nervous system through PD-1. In fact, B7-H1−/− B cells cotransferred were unable to prevent the inflammation (Fig. 5A). Therefore, in the current model B cells appear to downregulate T cell-induced necrotic inflammation in the liver by a B7-H1-dependent but IL-10-independent mechanism. It is important to note that T cell expansion in the liver was similar in all groups despite of the protection from B cells cotransferred (Fig. 5B), suggesting that the extent of T cell expansion/accumulation is not necessarily correlated with the disease progression.

Is B cell-mediated protection solely cell-to-cell interaction between T cells (and/or APCs) and B cells? Or alternatively, does it involve other soluble factor produced by B cells? Transfer of serum from ‘resistant’ TCRβδ−/− recipients was unable to reverse the inflammatory responses (Fig. 5C). To further investigate this possibility B cells isolated from MHCII−/− mice were used. Strikingly, MHCII−/− B cells had little impact on attenuating inflammation (Fig. 5D). Therefore, a direct interaction between H2M−/− T cells and MHCII+ B cells appears to be essential to upregulate B7-H1 on B cells possibly via IFNγ produced by activated T cells and thus to prevent the inflammation. Indeed, B cells cotransferred significantly upregulated surface B7-H1 expression (data not shown).

### The role of IFNγ signaling in liver pathogenesis

**Figure 6:**
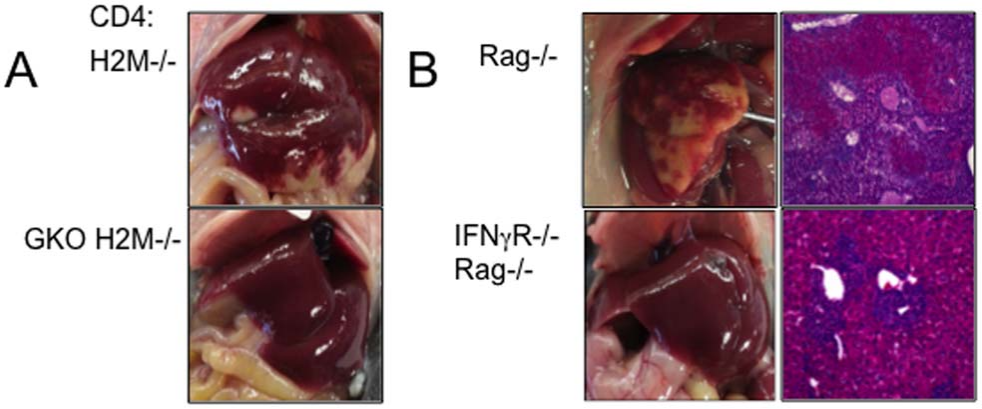
IFNγ is a key mediator of liver inflammation. (A) H2M−/− and GKO H2M−/− naïve CD4 T cells were adoptively transferred into Rag−/− recipients. (B) H2M−/− naïve CD4 T cells were adoptively transferred into Rag−/− and IFNγR−/− Rag−/− recipients. Liver pathology was determined at 7 days post transfer. Representative pictures from three independent recipients are shown.

H2M−/− CD4 T cells mediating unusual necrotic inflammation specifically in the liver primarily produce IFNγ during spontaneous proliferation. To examine whether IFNγ produced by H2M−/− CD4 cells mediate the inflammation, we transferred IFNγ−/− H2M−/− CD4 T cells into Rag−/− mice. As shown in Fig. 6A, the lack of IFNγ production by T cells completely abolished the pathogenicity. Likewise, IFNγR−/− Rag−/− recipients of WT H2M−/− CD4 T cells did not develop inflammation (Fig. 6B). Therefore, these results strongly suggest that IFNγ produced by activated T cells is a direct mediator of the inflammation.

## Discussion

In this study, we demonstrate an unusual spontaneous proliferation and the subsequent development of necrotic inflammation in the liver when H2M−/− CD4 T cells expressing limited repertoire diversity were used. CD4 T cells selected in the absence of H2M molecules acquire reactivity against self-MHCII:peptide complexes, the response leading to hyper-proliferative response even in coculture with syngeneic APCs [8], [10]. We investigated spontaneous proliferation and the subsequent development of intestinal inflammation using naïve H2M−/− CD4 T cells with the goal of evaluating the role of limited T cell repertoire diversity in developing colitogenic effector cells. Spontaneous proliferation of H2M−/− naïve T cells was greatly enhanced compared to that of WT T cells. Given the extent of spontaneous proliferation generally found in Rag−/− system and given the hyper-reactivity of H2M−/− T cells, this was somewhat expected. Unexpectedly, however, the recipients of H2M−/− T cells rapidly developed wasting disease with severe weight loss and high mortality. The wasting disease was not associated with intestinal inflammation typically seen in WT T cell recipients. Instead, the recipients developed acute hepatocellular necrotic inflammation, a response dependent on IFNγ and prevented by the presence of B cells. Because of the key role of T cells, T cell-derived IFNγ, and B cells in the pathogenesis, this may represent a useful in vivo model system to examine T cell induced liver inflammation [18].

Both T cell proliferation and liver inflammation require T cell interaction with MHCII molecules, since neither is found in MHCII−/− recipients (data not shown). Utilizing mixed bone marrow chimeras, we demonstrated the importance of MHCII expressed on bone marrow-derived cells, although the precise cell types expressing MHCII and presenting antigens remain to be determined. WT naïve CD4 T cells displaying diverse repertoire are induced to undergo proliferation only after interacting with MHCII+ DCs [14]. By contrast, it is possible that H2M−/− CD4 T cells are endowed with lower threshold for activation by non-DC MHCII+ cells including Kupffer cells. Alternatively, H2M−/− cells initially activated by DCs subsequently acquire hyper-reactivity with non-professional APCs including Kupffer cells and/or B cells. We favor this possibility because we observed that H2M−/− T cells transferred into MHCII−/− Rag−/− recipients failed to induce necrotic inflammation even after adoptive cotransfer with MHCII+ DCs (Do and Min, unpublished observation), an approach sufficient to induce CD4 T cell proliferation [19].

Our study also demonstrates a key role of B cells in preventing the hepatocellular necrosis. Unlike other B cell-mediated immune suppression typically mediated by IL-10-producing B cells (also known as regulatory B cells, [20]), B cells were capable of mediating protection via an IL-10-independent mechanism. Interestingly, B7-H1 expression on B cells seems critical for the action as reported in stroke model [17]. Based on the fact that B cell expression of MHCII is essential for protection, interaction between H2M−/− CD4 T cells and MHCII+ B cells may induce regulatory activity in B cells. We previously reported that MHCII molecules expressed on B cells do not induce CD4 T cell spontaneous proliferation [14]. Since TCR-MHCII interaction alone is not likely to induce regulatory activity by B cells, additional pathway(s) through costimulation and/or soluble factors may be involved.

What makes liver specific inflammation in this model? We initially hypothesized that this may resemble autoimmune hepatitis, which can be induced by an antigen specific T cells transferred into the liver specific antigen expressing transgenic mice [21]. However, when we compared the clonality of T cells activated in liver draining lymph nodes with those activated in other lymphoid tissues, we were unable to identify differences in the clonality. Moreover, T cells recovered from the inflamed liver still expressed highly diverse polyclonality. These results are not in favor of the possibility that some H2M−/− T cells are specific for liver antigens. However, it is important to point out that certain Vβ+ T cells do not mediate the pathology and that some Vβ+ T cells induce severe inflammation. Therefore, the relationship between H2M−/− T cell activation and inflammation in the liver remains to be examined.

In summary, the findings that H2M−/− CD4 T cells induce acute hepatocellular necrosis after transfer into B cell-deficient lymphopenic mice potentially offer a new animal model to study the pathogenesis of acute liver necrosis potentially triggered by adaptive immunity. Our results also provide a new in vivo model to study B cell-mediated immune regulation that is mediated by B7-H1 but not by IL-10. To the best of our knowledge, this is the first animal model that may open a new opportunity to study T cell-induced inflammation specifically in the liver and to gain insights into B cell-mediated immune regulation.

## Methods

### Animals

C57BL/6, B6 Rag1−/−, B6 TCRβδ−/− mice were purchased from the Jackson Laboratory (Bar Harbor, ME). B6 MHCII−/− Rag−/−, B6 IFNγR−/− Rag−/−, B6 Thy1.1 GKO H2M−/−, and B6 Thy1.1 H2M−/− mice were bred at the SPF animal facility of the Lerner Research Institute. IL-10−/− and B7-H1−/− mice were kindly provided by Drs. Stephen A Stohlman and Cornelia Bergmann (Department of Neuroscience, Cleveland Clinic Foundation), respectively. All the animal procedures reported in this study were carried out in accordance with the recommendations in the Guide for the Care and Use of Laboratory Animals of the National Institutes of Health. The Institutional Animal Care and Use Committee of the Cleveland Clinic Foundation approved this study (Animal Welfare Assurance No. A3047-01. IACUC protocol No. 0877). All animals were euthanized by CO2 inhalation followed by cervical dislocation.

### Cell sorting and adoptive transfer

Lymph node (LN) naive CD4 T cells were obtained from the peripheral LN (axillary, cervical, and inguinal LN) and mesenteric LN. CD4 T cells were purified by negative selection as previously described [14]. CD44^l^°^w^ CD25- naive CD4 T cells were further sorted using a FACSAria cell sorter (BD Bioscience, San Jose, CA). 1×10^6^ naive T cells were transferred alone or in combination with 5×10^6^ FACS sorted CD19+ B cells into Rag−/− and B6 TCRβδ−/− mice. After T cell transfer, mice were sacrificed to measure donor cell expansion. In experiments to deplete B cells, mice were intravenously injected on Day −1 with 250 µg anti-mouse CD20 Ab (18B12) and isotype-matched control Ab (2B8), which were provided from Biogen Idec.

### Bone marrow reconstitution

Bone marrow chimeras were generated as previously reported [14].

### FACS analysis

Cells were stained with anti-CD4 (RM4-5), anti-CD19 (1D3), anti-CD25 (IL-2Rα, p55), anti-CD44 (IM7), anti-Thy1.1 (HIS51), anti-IL-17A (eBio17B7), anti-IFNγ (XMG1.2), anti-MHCII (M5/114.15.2), anti-B7-H1 (MIH5) and anti-IL10 (JES5-16E3) (all Abs from eBioscience). Cells were acquired using a FACS LSR II (BD Biosciences) and analyzed using a FlowJo software (Treestar, Ashland, OR). To measure the frequency of cytokine producing CD4 T cells, cells were stimulated with PMA (10 ng/ml) and Ionomycin (1 µM) for 4 hrs in the presence of 2 µM Monensin (Calbiochem, San Diego, CA) during the last 2 hrs. Cells were immediately fixed with 4% paraformaldehyde, permeabilized, and stained with fluorescence conjugated antibodies.

### Immunohistochemistry

Liver tissue was fixed in 10% acetic acid/60% methanol solution, and H&E stained. For immunohistochemistry paraffin embedded liver sections were incubated overnight at 4°C in primary anti-CD3 (ab5690, abcam, Cambridge, MA), followed by secondary biotinylated goat anti-rabbit IgG (Jackson Immuno Research, West Grove, PA). Vectastain Elite ABC Kit and DAB (Vector Laboratories, Burlingame, CA) was used for color development, and processed for mounting using Clarifier I, Bluing Solution, Clear-Rite 3 and Mounting Media (all Richard-Allan, Kalamazoo, MI).

### Statistical analysis

Statistical significance was determined by the Mann-Whitney *U* test using the Prism 5 software (GraphPad, La Jolla, CA). A p value of <0.05 was considered statically significant. Kaplan-Meier survival curves were generated using the Prism 5 software, and log-rank testing was used to determine significance. Of note, H-2M−/− T cell recipients displayed 100% mortality by 21 days post transfer. WT T cell recipients showed 0% mortality until 42 days post transfer at which they typically develop T cell-mediated colitis.

## Supporting Information

Checklist S1 **ARRIVE Guidelines Checklist.**
(PDF)
